# Characterizing Macroporous Ion Exchange Membrane Adsorbers for Natural Organic Matter (NOM) Removal—Adsorption and Regeneration Behavior

**DOI:** 10.3390/membranes14060124

**Published:** 2024-05-27

**Authors:** Jon Wullenweber, Julia Bennert, Tomi Mantel, Mathias Ernst

**Affiliations:** 1 Institute for Water Resources and Water Supply, Hamburg University of Technology, Am Schwarzenberg-Campus 3, 21073 Hamburg, Germany; 2 DVGW Research Centre TUHH, Am Schwarzenberg-Campus 3, 21073 Hamburg, Germany

**Keywords:** membrane adsorber, ion-exchange membrane, adsorption, natural organic matter, NOM removal, water treatment, adsorptive membranes

## Abstract

Addressing the characterization of Natural Organic Matter (NOM) removal by functionalized membranes in water treatment, this study evaluates the effectiveness of two commercial ion-exchange membrane adsorbers: Sartobind^®^ Q (with quaternary amines) and D (with tertiary amines). Using Suwannee River NOM (SRNOM) as a surrogate, Langmuir adsorption isotherms revealed maximum capacities (Q_max_) of 2966 ± 153 mg C/m^2^ and 2888 ± 112 mg C/m^2^, respectively. Variations in flux from 50 to 500 LMH had a minimal impact on breakthrough times, proving low diffusion limitations. The macroporous (3–5 µm) functionalized cellulose-based membranes exhibited high permeabilities of 10,800 L/(h m^2^ bar). Q maintained positive zeta potential vs. pH, while D’s zeta potential decreased above pH 7 due to amine deprotonation and turning negative above an isoelectric point of 9.1. Regeneration with 0.01 M NaOH achieved over 95% DOC regeneration for Sartobind^®^ D, characterizing reversibility through a pH-swing. Cyclic adsorption showed that Q maintained its capacity with over 99% DOC regeneration, while D required acidic conditioning after the first regeneration cycle to mitigate capacity reduction and re-deprotonate the adsorber. These results have demonstrated the potential suitability of adsorber membranes, designed originally for biotechnological purposes, for the possible removal of disinfection byproduct precursors in drinking water treatment.

## 1. Introduction

Natural Organic Matter (NOM) is a complex mixture of carbon-based compounds present in surface and ground waters [1]. It originates from the decomposition of plant and animal residues, as well as from microbial synthesis [2]. While NOM in drinking water is not harmful per se, it poses an ongoing challenge in drinking water treatment regarding unwanted color and taste [1], turbidity, disinfection-byproduct (DBP) formation potential [3,4] and possible microbial growth [5,6]. Consequently, the effective removal of relevant NOM fractions from source waters is a crucial aspect of water treatment processes in regard to ensuring the safety and quality of drinking water. Conventional methods for NOM removal include coagulation−flocculation, adsorption on activated carbon, and membrane filtration. However, these methods have limitations, such as incomplete removal of NOM, high operational costs and/or generation of secondary waste. Most of the aquatic NOM is negatively charged at neutral pH [7]; therefore, ion-exchange (IEX) offers great potential in removing NOM from drinking water sources. Anion exchange in NOM removal has extensively been studied but is often limited by factors like slow pore diffusion, pressure drops and low flow rates [7,8,9,10]. In addition, the use of neutral salts for regeneration, brine deposition and recycling are relevant problems for large-scale applications.

Adsorption refers to the process of removing or adhering molecules or substances from one phase, such as water, to a surface, such as a membrane. This process is widely utilized in water treatment [11], such as in the removal of heavy metals [12] or NOM [13]. Adsorptive membranes (AM), a special type of mostly macroporous membranes, are designed to integrate the steric retention of conventional membrane filtration with the properties of adsorptive materials, offering the advantage of high selectivity coupled with high adsorption capacities [14,15]. Membrane adsorbers, a subclass of adsorptive membranes, are configured to function more like a traditional adsorbent but within a membrane filtration configuration, typically consisting of a porous base matrix that is functionalized with adsorptive materials/groups [16].

Ion-exchange membrane adsorbers present an innovative approach to overcome some of the limitations associated with traditional adsorbents or IEX resins. The macroporous pores of the membranes contain IEX groups (e.g., positively or negatively charged) that selectively bind ions or charged molecules. Therefore, diffusion limitations typically present with beads, and resins are significantly reduced, as the convective mass transport takes place through the functionalized pores [16]. These membrane adsorbers combine the high surface area of membranes with the selective adsorption properties of IEX-materials, leading to rapid kinetics and potential high NOM removal efficiencies. The use of membrane adsorbers is a niche technology and has long been overlooked [17]. They are primarily used for IEX chromatography within a membrane format and find applications in bioprocessing and pharmaceutical manufacturing for the separation of proteins, nucleic acids and other biomolecules with charge-based separations [17,18]. Studies have explored both commercial and custom-fabricated porous anion-exchange membranes for various applications in water treatment, like the removal of anionic dyes [19,20,21], heavy metals [15,22,23,24] or PFAS [25].

The successful application of membrane adsorbers in the removal of anionic compounds underscores their potential in the removal of NOM, which predominantly carries a negative charge. This paper investigates the application of two commercially available ion-exchange membrane adsorbers on the removal of NOM. The choice for these membranes was based on their successful application in biotechnology, where it has been shown that they possess very high binding capacities for anionic bioproteins such as Bovine Serum Albumin (BSA) [26]. Anion exchangers (AEX) are classified into two categories: strong basic and weak basic. Weak basic AEX are characterized by their ability to protonate and deprotonate, making their positive charge dependent on the pH level [27]. Studies have shown that these two categories of AEX differ in their capability for removing NOM [7,8]. The study aims at elucidating the adsorption behavior of two types of anion exchange membrane adsorbers—weak basic and strong basic—depending on varying water chemistry and residence time while also exploring two regeneration mechanisms: a pH-Swing technique and a a neutral salt method.

## 2. Materials and Methods

### 2.1. Membranes

The membranes utilized in this study are commercially available and constructed from a reinforced stabilized cellulose base material to which IEX ligands are covalently bound with a nominal pore size ranging from 3–5 µm: Sartobind^®^ D, characterized as a weak basic ion-exchange membrane functionalized with diethylamines (R-CH_2_-N(C_2_H_5_)_2_) and Sartobind^®^ Q, with strong basic IEX properties functionalized with quaternary amines (R-CH_2_-N+(CH_3_)_3_) (both Sartorius Stedim Biotech GmbH, Göttingen, Germany). Characteristics of the membrane are displayed in Table 1.

### 2.2. Membrane Characterization

The pure water permeability (PWP) of the membranes was determined using a lab-scale system with an Amicon^®^ dead-end cell (Model 8200, Merck Millipore, Burlington, MA, USA). The membrane samples had an active area (A) of 28.7 cm^2^ with a diameter of 6.2 cm. Prior to measurement, the membranes were pre-conditioned by immersing them into deionized (DI) water before being thoroughly rinsed. The pressure (Δp) was systematically changed to three different levels: 0.6, 1 and 1.2 bar. After equilibration, permeate was collected for 1 min (Δt) and weighed to determine the volume (ΔV). This process was conducted in triplicates for each pressure condition. The resulting PWPs were determined by applying Equation (1):

(1) $$Jw=\frac{\Delta V}{\Delta t\cdot \Delta p\cdot A}$$

For membrane contact angle (CA) assessment, the captive bubble method was employed in DI water. The shape of the water droplets was captured through imaging. Membrane samples measuring 20 × 10 mm were secured in a holder, and air bubbles were introduced onto the membrane surface using an inverted J-shaped needle, with measurements performed in triplicates. The resulting CAs were determined using the Software Version Surftens 4.5 (OEG GmbH, Frankfurt an der Oder, Germany).

Surface morphology was analyzed using scanning electron microscopy (SEM) imaging with a Zeiss Leo Gemini 1530 (Carl Zeiss AG, Oberkochen, Germany).

The zeta potential measurements were performed on a SurPASS electrokinetic analyzer (Anton Paar GmbH, Graz, Austria) and an adjustable gap cell to accommodate membrane surface samples with a rectangular size (20 × 10 mm). The same gap cell was used for zeta potential measurements of both membranes. The average thickness of the samples corresponded to the membrane thickness of approximately 250 µm, as listed in Table 1. The gap height was manually set to 100 ± 10 µm and controlled via differential pressures and flow rates. The pH was initially adjusted to alkaline conditions (pH 11) using sodium hydroxide (NaOH). Measurements were conducted using the streaming current method in auto-titration mode, with hydrochloric acid (HCl) as the titrating acid, targeting an endpoint of pH 3. To avoid the adsorption of CO_2_ during the measurements, any air within the system was effectively removed by purging with nitrogen gas. The methodology is described in detail elsewhere [29].

ATR-FTIR spectra were acquired employing a Vertex 70 spectrometer (Bruker Optik GmbH, Ettlingen, Germany). This spectrometer was outfitted with a single-bounce diamond ATR insert (MIRacle, Pike Technologies) and featured a liquid-cooled MCT detector. Flushing was carried out with dry, carbon dioxide-free air before analysis. Spectral data were gathered utilizing 64 scans per spectrum, from 4000 cm^−1^ to 600 cm^−1^, with the OPUS 7.0 software (Bruker Optik GmbH, Ettlingen, Germany).

### 2.3. Feed Solution

Suwannee River Natural Organic Matter (SRNOM) isolate (2R101N), a NOM of representative aquatic origin, was obtained from the International Humic Substances Society (IHSS, USA). This choice was based on its relevance and comparability to the existing literature [30]. The SRNOM was dissolved over one week, with gentle stirring being used to ensure complete dissolution. Subsequently, the solution underwent prefiltration through a 0.45 µm filter to eliminate undissolved compounds and larger particulate matter [1]. The specific ultraviolet absorbance (SUVA) was determined to be 3.86 ± 0.09 L/(mg·m), as detailed through a calibration curve provided in Figure S1. NaOH, HCl, sulfuric acid (H_2_SO_4_) and sodium chloride (NaCl) (all obtained from Carl Roth GmbH + Co KG, Karlsruhe, Germany) were employed for adjustments and variations in pH as well as ionic strength.

### 2.4. NOM Analytics

To determine the organic carbon content within the samples, measurements of dissolved organic carbon (DOC) were carried out using a Shimadzu TOC analyzer instrument. Additionally, Ultraviolet-254 (UV254) measurements were conducted employing a spectrophotometer (DR5000, Hach Lange GmbH, Düsseldorf, Germany), where the absorbance of ultraviolet light at the wavelength of 254 nm was assessed to estimate the concentration of DOC in the water samples [31].

Liquid Chromatography-Organic Carbon Detection (LC-OCD) was employed to further characterize the DOC in the water. This technique involved the separation of organic compounds using liquid chromatography coupled with organic carbon detection, providing detailed insights into the composition and structure of the chromatographable dissolved organic carbon (CDOC). An extensive procedure is described elsewhere by Huber et al. (2011) [32].

For fluorescence analysis, the water samples were subjected to spectrofluorometric measurements using an Aqualog fluorescence spectrophotometer (HORIBA, Osaka, Japan). Emission-excitation matrices (EEMs) were recorded to characterize the properties of fluorescent dissolved organic matter (fDOM), offering information on its origin, composition and structural characteristics.

### 2.5. Adsorption Experiments

#### 2.5.1. Static Adsorption

**Figure 1:**
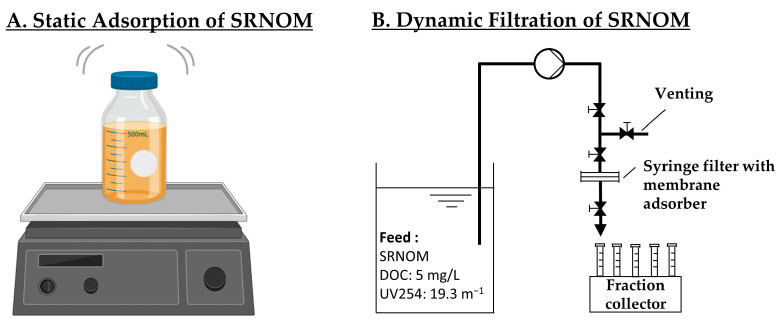
Experimental set-ups for (**A**): static adsorption of SRNOM on an orbital shaker (created with BioRender.com); (**B**): dynamic filtration of SRNOM with a peristaltic pump and fraction collector.

Adsorption isotherms were gathered to assess the adsorption characteristics. Membrane cutouts measuring 1 cm^2^ (A) in size were immersed in 500 mL (V) of the feed solution at five discrete concentrations (0.5, 1, 2.5, 5, 10 mg/L DOC) (c_0_) at a pH of 7. Adsorption was conducted on orbital shakers set at 150 rpm for 7 days, as displayed in the experimental set-up shown in Figure 1A. Upon completion of the experiments, the concentrations (c_e_) of DOC in the solution were quantified to determine the extent of adsorption. Each experiment was conducted in duplicates, and the analyses were performed in triplicates. Adsorption capacity (Q_e_) was calculated using Equation (2):

(2) $$Q_{e}=\frac{{(c}_{0}-c_{e})V}{A}$$

The Langmuir model, which assumes monolayer adsorption on a homogeneous surface with finite identical sites, is represented by Equation (3), where Q_e_ is the amount adsorbed at equilibrium, C_e_ is the equilibrium concentration of the adsorbate, Q_m_ is the maximum adsorption capacity, and K_L_ is the Langmuir constant related to the affinity of the binding sites.

(3) $$Q_{e}=\frac{Q_{\max}K_{L}C_{e}}{{1+K}_{L}C_{e}}$$

The Freundlich model, on the other hand, is an empirical equation that describes adsorption on heterogeneous surfaces and the possibility of multilayer adsorption. It is given by Equation (4), where K_f_ and n are Freundlich constants indicative of the adsorption capacity and the adsorption intensity, respectively.

(4) $$Q_{e}=K_{f}{C_{e}}^{n}$$

The R^2^ value and chi-square χ^2^ value derived from Equations (5) and (6), respectively, were calculated to assess the goodness-of-fit of the models to the experimental data [33]. Where Q_e,exp_ represents the DOC uptake at equilibrium, Q_e,cal_ represents the calculated value from the models and Q_e,mean_ represents the average of the Q_e,exp_ values.

(5) $$R^{2}=1-\frac{{\sum (Q_{e,\exp}-\;Q_{e,\mathrm{cal}})}^{2}}{{\sum (Q_{e,\exp}-\;Q_{e,\mathrm{mean}})}^{2}}$$

(6) $$\chi^{2}=1-\frac{{\sum (Q_{e,\exp}-Q_{e,\mathrm{cal}})}^{2}}{Q_{e,\mathrm{cal}}}$$

#### 2.5.2. Dynamic Adsorption

It is essential to emphasize that membrane adsorption is inherently a dynamic process when applied in flow-through mode, and the time/treated volume until a breakthrough occurs holds significant importance, as it is a key parameter for process design in practical applications. Hence, dynamic breakthrough experiments were conducted utilizing a peristaltic pump (ISM931A, Ismatec, Wertheim, Germany) and a polycarbonate syringe filter holder (Sartorius, Göttingen, Germany) as a membrane cell (filtration area 3.8 cm^2^) (see Figure 1B). To ensure even distribution of the feed, a feed spacer mesh was placed in the feed channel. Before the experiments, membranes were extensively flushed with DI water and de-aerated. Permeate collection was facilitated by an automated LAMBDA OMNICOLL^®^ fraction sampler (LAMBDA Laboratory Instruments, Baar, Switzerland). To investigate the influence of the flux and water chemistry (pH and ionic strength) on the dynamic adsorption in each set of experiments, one of the three parameters was varied, while the others remained constant at their default values (Flux of 500 LMH, no addition of NaCl and a pH of 7). The SRNOM feed solution had a DOC concentration of 5 ± 0.1 mg/L and a UV254 of 19.3 ± 0.1 m^−1^. Flux variation was explored across four distinct flux rates (50, 100, 250 and 500 LMH) and ionic strength investigation involved the addition of NaCl at concentrations of 0.1, 1 and 10 mmol/L. The pH was systematically varied at pH levels of 4.5, 7, 9 and 11. All experiments were run in duplicates and analyzed in triplicates.

### 2.6. Regeneration

#### 2.6.1. Batch Desorption

**Figure 2:**
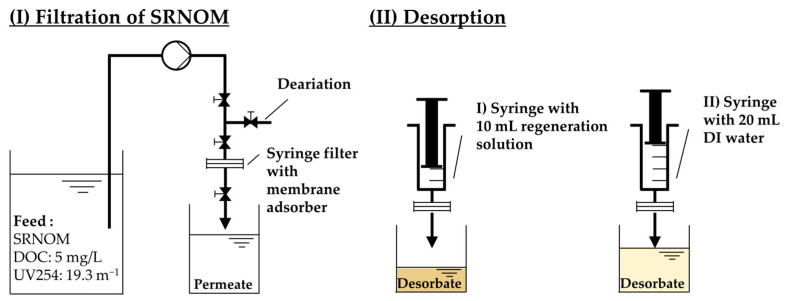
Experimental set-up for regeneration experiments of SRNOM using different regeneration solutions.

To investigate the potential of pH-swing-induced regeneration of the spent adsorber membranes, regeneration experiments were conducted with varying molarities of NaOH, thereby controlling the pH. In contrast to this approach, a comparison was made with plain IEX regeneration involving NaCl and hydrochloric acid (HCl). Regeneration experiments were conducted as shown in Figure 2, initiating with the filtration of 250 mL of 5 mg/L SRNOM feed solution (pH = 7) through the membrane (flux = 500 LMH). For regeneration, solutions of 1 M HCl, 1 M NaCl and NaOH concentrations ranging from 0.1 mM to 1 M were employed, with 10 mL carefully pushed through the membrane using a syringe, followed by a 20 mL flush with DI water to remove any desorbed residuals. The 10 mL regenerate volume was determined through preliminary experiments, as depicted in Figure S2 in the Supplementary Information. All experiments were conducted in duplicate. The regeneration efficiency of DOC (%R_DOC_) was calculated using Equation (7), with c_DOC,i_ and V_i_ representing the concentration and volume of the permeate (P) and desorbate (D), respectively:

(7) $$\%R_{\mathrm{DOC}}=\frac{c_{\mathrm{DOC},D}\cdot V_{D}}{c_{\mathrm{DOC},P}\cdot V_{P}}$$

#### 2.6.2. Cyclic Adsorption and Regeneration

The same experimental setup for dynamic adsorption as described in Section 2.5.2 was employed for cyclic adsorption. In this investigation, 400 mL of the feed solution (till an expected breakthrough of approx. 80 %) was initially filtered under the default conditions (DOC 5 mg/L, UV254 19.3 m^−1^, pH 7, flux 500 LMH).

Following the filtration process, the regeneration procedure, as detailed in Section 2.6.1, was executed, utilizing a 1 M NaOH solution. Subsequently, the membranes were de-aerated to prepare for the next adsorption cycle. A total of five adsorption-regeneration cycles were conducted. These experiments were performed in duplicate. This cyclic adsorption and regeneration study aimed to assess the membrane’s suitability for repeated use and its overall efficiency in removing NOM from the feed solution.

## 3. Results and Discussion

### 3.1. Membrane Characterization

#### Surfase Morphology

**Figure 3:**
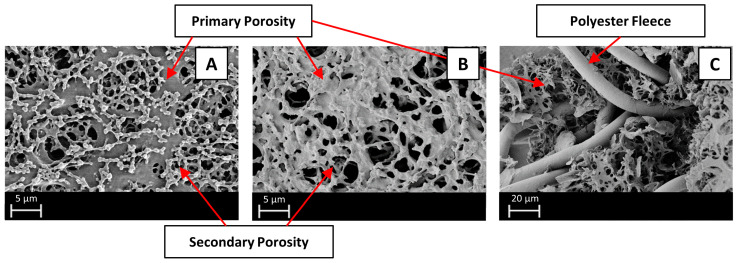
SEM images of: (**A**) Sartobind^®^ Q; (**B**) Sartobind^®^ D; (**C**) cross-sectional perspective.

SEM images of Sartobind^®^ Q and D, alongside a cross-sectional perspective, are presented in Figure 3. These images reveal a reinforced, cross-linked cellulose structure. Both membranes exhibit a primary porous structure that is achieved through the process of evaporative casting. The pore size, as specified by the manufacturer (3–5 μm), aligns well with the primary structure observed in the SEM images. Furthermore, the remaining pores, constituting the secondary porous structure, are filled with residual casting solution. This phenomenon results in a refinement of porosity, ultimately leading to a higher adsorption area on the membrane surface [34]. Both membranes thus fall in the range of microfiltration (MF) membranes, with pore sizes typically ranging from 0.1 to 5 μm. As such, it can be classified as an MF membrane, sharing the advantage of high fluxes achievable at low pressures. Without functionalization, MF membranes’ efficacy in NOM removal is negligible due to the larger pore size compared to the size of NOM, as conventional membrane filtration primarily relies on steric retention and physical barriers [35]. The pore sizes are larger than the largest SRNOM fractions to be removed [36]. Thus, removal is focused solely on adsorptive processes, limiting external fouling through cake layer formation. This design ensures high fluxes and low pressure drops.

In the cross-section in Figure 3C, an additional element of reinforcement is visible in the membrane structure. A coarse structure composed of polyester fleece is incorporated for mechanical stability, enhancing the robustness and durability of the membranes. Considering the regenerated cellulose-based backbone of the membrane, additional mechanical support is required to withstand operational stresses, like particulate matter. As further elucidated by Wang et al., the mechanical stability of the membranes is significantly attributed to the thick polyester fibers (~10 μm diameter) that are concentrated in the middle section of the membrane [37].

**Figure 4:**
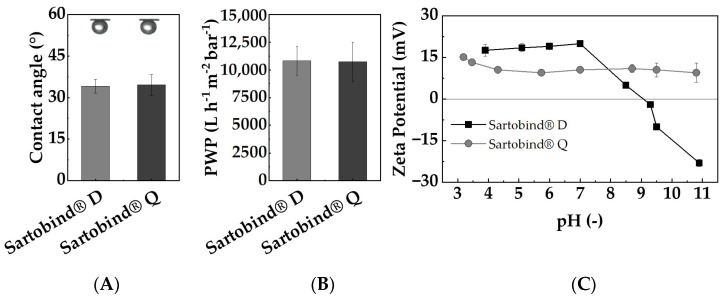
Membrane characteristics of Sartobind^®^ D and Q: (**A**) contact angle determined using captive bubble method; (**B**) pure water permeability determined across three pressures (0.6, 1, 1.2 bar); (**C**) zeta potential determined through titration from pH 11 to pH 3 using the streaming current method over a pH range of 3–11 using the streaming current method.

Figure 4A displays the CAs of Sartobind^®^ D and Q. The regenerated cellulose membranes yielded angles of 34.1 ± 2.5 ° and 34.6 ± 3.7 °, respectively. The consistent CA indicates that the distinct functionalization with tertiary and quaternary amines does not alter the hydrophobicity to different degrees. These results are comparable to findings on regenerated cellulose membranes, which are not, or only to a slight degree, modified [38,39], suggesting minimal impact on the inherent hydrophilic nature of the cellulose base material. This could be attributed to the preservation of hydrogen bonding sites or the overall polar nature of the cellulose backbone [40], which might dominate the surface interaction with water despite the presence of hydrophobic amine groups. Its hydrophilicity ensures high permeabilities for aqueous solutions, facilitating efficient fluid transport. Moreover, the low CA makes the membrane prone to adsorptive internal fouling, which aligns with the intended adsorption mechanism, allowing for the targeted adsorption of NOM on its surface.

The PWPs of both membranes are displayed in Figure 4B, with both membranes exhibiting PWPs of approximately 10,800 L/(h m^2^ bar). This observation is in line with the hydrophilic nature and substantial pore sizes of the membranes. Moreover, at neutral pH, the PWP remains consistent for both membranes despite their different functionalization, suggesting that variations in swelling or other effects due to functionalization do not alter their PWPs to different degrees.

Given that the predominant portion of NOM is negatively charged under neutral conditions, a desirable characteristic for effective adsorptive removal is a positively charged membrane adsorber surface. In Figure 4C, the recorded zeta potentials across a pH range from 3 to 11 are shown. Sartobind^®^ Q, functionalized with quaternary amines, exhibits a consistently positive surface potential across the entire pH range. Notably, quaternary amines, by their intrinsic charge, cannot be protonated even at alkaline pH levels. In contrast, Sartobind^®^ D, functionalized with diethylamine, displays a decline in surface charge, starting from a pH value of 7 and an isoelectric point (IEP) at pH 9.1. Above the IEP, it exhibits a negative charge. The behavior of Sartobind^®^ D can be attributed to the weak basic properties of diethylamine. As the pH increases, more functional groups deprotonate, consequently losing their positive charge. The determined IEP aligns with the pKa (9.5) value for cellulose−diethylamine provided by the manufacturer. These findings highlight the fundamental suitability of both membranes for applications in drinking water treatment within the common pH range of 6.5–8.5.

The ATR-FTIR spectra, presented in Figure S3, exhibit peaks at 3364 cm^−1^ and 1650 cm^−1^, which potentially correspond to the stretching vibrations of amines and amidines (N=C–N), indicating possible functionalization [41]. The signal of the bands at 3364 cm^−1^ was more pronounced for the quaternary amines of the Sartobind^®^ Q than for the tertiary amines of the Sartobind^®^ D. A similar trend is also described in the literature [42]. Due to the absence of a comparative analysis with the pristine base cellulose material used by the manufacturer, these peak assignments are provisional, yet they point to significant chemical alterations in the membrane structure.

### 3.2. Adsorption Characteristics

#### 3.2.1. Comparative Analysis of NOM Removal

Distinct maximum removal rates for both membranes were observed by assessing UV254, DOC, LC-OCD and fluorescence spectroscopy analysis. The removal rates for UV254 active compounds and DOC were higher with Sartobind^®^ D, achieving 95.6 ± 0.2% and 83.7 ± 0.3%, respectively, compared to 94.3 0.3% and 80.4 0.5% with Sartobind^®^ Q. The higher UV254 removal rate compared to DOC for both membranes indicates a preferred removal of aromatic compounds, as UV254 is a more specific indicator for these types of molecules, which is consistent with the literature on adsorptive membranes and IEX processes for NOM removal [9,43].

**Figure 5:**
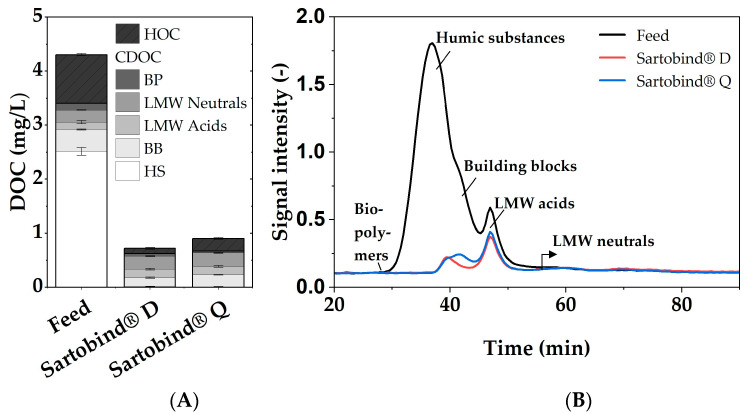
(**A**) Fractionation of HOC and CDOC and (**B**) LC-OCD chromatograms of the feed and permeates of Sartobind^®^ D and Q.

The chromatographic analysis further supports these findings, as illustrated in Figure 5, which presents the fractionation of DOC by LC-OCD. The chromatograms reveal that, while the overall CDOC was removed to a similar extent by both membranes, the specific fractions within this CDOC were targeted differently. The largest fraction, humic substances (HS), saw a removal efficiency of almost 100% with both membranes. The fraction of building blocks (BB), as evident in the chromatogram, is better removed by the Sartobind^®^ D than Q, with 61% to 46% removal, respectively. The removal rates for biopolymers (BP) cannot be determined, as these compounds are not present in the NOM model water. Since both membranes removed approximately 80% of the CDOC, the difference in total DOC removal can not only be explained by the difference in BB removal but also by the non-chromatographable fraction of organic carbon, defined by Huber et al. (2011) [32] as hydrophobic organic carbon (HOC). Sartobind^®^ D demonstrated a notably higher HOC removal efficiency of 89 %, compared to 74% by Sartobind^®^ Q. This discrepancy could be attributed to the different charge densities of the tertiary and quaternary amines, affecting their affinity for hydrophobic DOC fractions [44].

Furthermore, the fluorescence excitation−emission matrix (EEM) analysis, depicted in Figure S4, aligns with the LC-OCD and UV254/DOC findings. The overall fluorescence intensity decrease was stronger for Sartobind^®^ D. Especially for peak M, which is indicative of humic-like substances [45], Sartobind^®^ D achieved an 84% reduction compared to 74% for Sartobind^®^ Q. This could be attributed to the difference in BB removal, which has similar characteristics to humics.

The substantial removal of HS by both membranes is associated with the strong negative charge these substances exhibit at neutral pH due to their phenolic and carboxylic functional groups [46,47]. This charge facilitates the affinity and subsequent adsorption of HS onto the membranes. Given that HS can account for up to 90% of the DOC in natural waters [2,48], the efficiency of these membrane adsorbers in removing HS underscores their versatility and potential effectiveness in a variety of water treatment contexts.

#### 3.2.2. Static Adsorption

**Figure 6:**
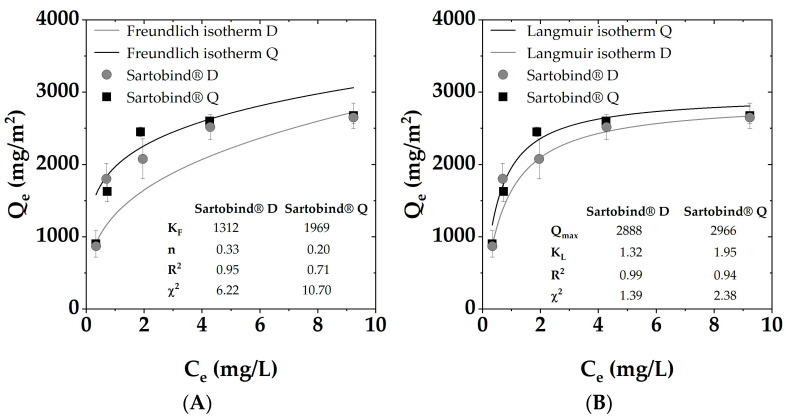
Adsorption isotherms for SRNOM removal via Sartobind^®^ D and Q with fitted (**A**) Freundlich model and (**B**) Langmuir model.

For both membranes, the Q_e_ was found to be similar at lower DOC concentrations. However, as the initial concentration exceeded 5 mg/L DOC, Sartobind^®^ D exhibited a notably higher adsorption capacity compared to Sartobind^®^ Q. The adsorption isotherms (displayed in Figure 6) were analyzed using both Langmuir and Freundlich models to understand the adsorption mechanisms and potential surface heterogeneity of the membranes. The DOC uptake was related to the membrane area (mg/m^2^).

For both membranes, the Langmuir model provided a better fit with R^2^ values of 0.99 for the D and 0.94 for the Q, respectively, suggesting monolayer adsorption onto a homogeneous surface with finite adsorption sites. The Freundlich model, which accounts for heterogeneous surface adsorption, also showed a commendable fit, with an R^2^ value of 0.95 for the Sartobind^®^ D and only 0.71 for the Q. Evaluating the χ^2^ values underlined the indication for the better fit of the Langmuir models with lower values for both membranes.

The Q_max_ values derived from the Langmuir isotherm were found to be in a comparable range, with 2888 ± 112 mg C/m^2^ for the Sartobind^®^ D and 2966 ± 153 mg C/m^2^ for the Sartobind^®^ Q.

### 3.3. Dynamic Adsorption

#### Influence of Flux

**Figure 7:**
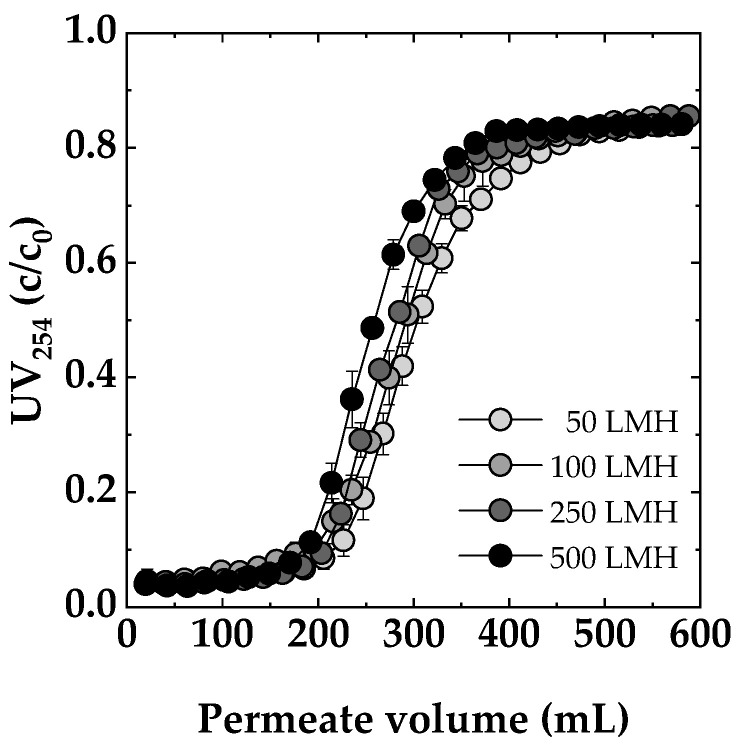
Impact of flux (50, 100, 250, 500 LMH) on dynamic breakthrough. Membrane: Sartobind^®^ D. Feed: SRNOM 5 mg/L DOC, pH 7, 0 mmol/L NaCl.

As shown in Figure 7, the dynamic breakthrough curves revealed distinct trends as a function of flux rate. At 50 LMH, the Sartobind^®^ D membrane adsorber demonstrated the most extended contact time, resulting in enhanced performance. This finding indicated that lower flux rates allowed for effective adsorption and prolonged interaction between the feed solution and the adsorbent ligands. Transitioning to a flux rate of 100 and 250 LMH, there was a slight reduction in performance compared to 50 LMH, but the decline was not substantial. This observation suggested that, even at higher fluxes, the membrane adsorber remained effective for NOM removal. Moving to higher flux rates, a more pronounced decrease in performance was observed. At 500 LMH, the breakthrough occurred approximately 10% earlier compared to 50 LMH. Nevertheless, the membrane adsorber still exhibited a reasonable level of efficiency. Similar trends were observed for a chitosan-based membrane adsorber in copper removal, where an earlier breakthrough of approximately 10% occurred after increasing the flux from 50 LMH to only 200 LMH. This phenomenon could be explained by the rate-limiting step shifting to the binding rate between the target solute and the adsorption sites on the membrane surface as the flow rate increases [49]. It is noteworthy that studies [26,50], which investigated the same membrane adsorbers used in this study for the adsorption of BSA, emphasized that these membranes are not harshly flow rate/flux dependent and have very similar results for the NOM removal dependency on flux investigated in this study. Compared to conventional adsorptive or ion exchange methods, the low flux dependency observed in these membrane adsorbers is a significant advantage.

### 3.4. Influence of Water Chemistry Parameters

In addition to examining the impact of flux, it was investigated how water chemistry parameters, specifically pH value and ionic strength, affect the removal of SRNOM.

#### 3.4.1. Ionic Strength

**Figure 8:**
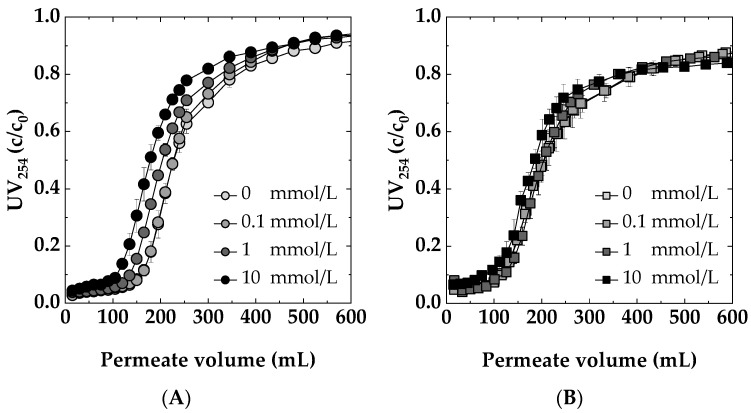
Impact of ionic strength at different NaCl concentrations: 0, 0.1, 1, 10 mmol/L on the dynamic breakthrough curves for: (**A**) Sartobind^®^ D and (**B**) Sartobind^®^ Q. Feed: 5 mg/L DOC, pH 7, Flux 500 LMH.

Figure 8A,B display the dynamic breakthrough curves for Sartobind^®^ Q and D, respectively, at varying NaCl concentrations. The results indicate that a very low salt concentration (0.1 mmol/L NaCl) neither enhances nor deteriorates the removal efficiency of UV254 active compounds by the membrane adsorbers. The comparable performance at concentrations of 0 and 0.1 mmol/L suggests that such minimal levels of NaCl have a neutral impact on the interaction between NOM and the adsorbers. However, at increased salt concentrations (1 and 10 mmol/L), a quicker breakthrough of NOM is observed for both membranes, indicating a decline in the adsorption efficiency. This is particularly evident in the weak basic Sartobind^®^ D, where the higher ionic strength likely leads to competitive interactions between chloride ions and NOM molecules for adsorption sites, resulting in decreased NOM removal efficiency. For Sartobind^®^ Q, the observed weaker performance at higher salt concentrations can be explained due to the predominant compromised coulombic interactions [51]. The decreased adsorption for Sartobind^®^ D can be attributed to two main factors. Firstly, according to Jones et al. [52], increased salt concentration diminishes electrostatic attraction between oppositely charged entities, reducing adsorption efficiency. Secondly, Mo et al. (2008) [53] demonstrated that higher ionic strength, as seen with 10 mmol/L NaCl, decreases the zeta potential of the membrane surface. An increase in ionic strength leads to the compression of the electrical double layer surrounding the charged membrane and dissolved organic molecules [54]. Furthermore, the presence of additional ions in the solution can enhance the shielding effect on the charged groups present on the membrane surface and the adsorbate molecules. This shielding neutralizes the interactions that typically favor adsorption, such as electrostatic attractions between oppositely charged sites or repulsions that prevent the approach of like-charged molecules.

#### 3.4.2. pH

**Figure 9:**
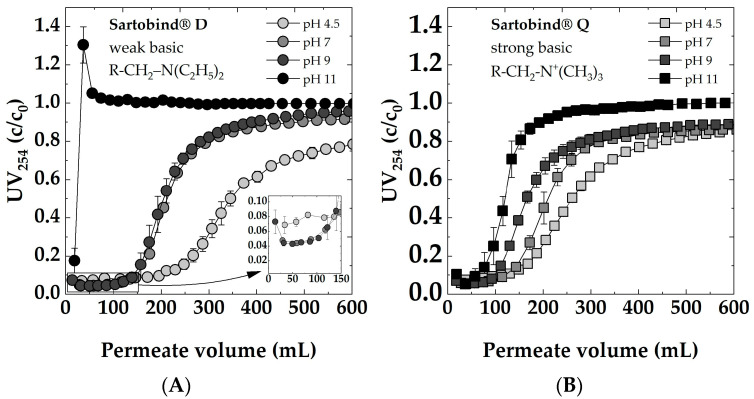
Impact of feed pH (4.5, 7, 9, 11) on dynamic breakthrough curves for: (**A**) Sartobind^®^ D and (**B**) Sartobind^®^ Q. Feed 5 mg/L DOC, 0 mmol/L NaCl, Flux 500 LMH.

To investigate the impact of varying feed pHs on adsorption behavior, dynamic breakthrough curves for both membranes are presented in Figure 9. Sartobind^®^ D, characterized by tertiary amine functional (Figure 9A), and Sartobind^®^ Q, featuring quaternary amine functional groups (Figure 9B), exhibited distinct pH-dependent filtration behaviors. Notably, both membranes displayed delayed breakthroughs and demonstrated greater overall removal efficiency at lower pH values. The observed behavior can be attributed to the charge characteristics of the membranes’ functional groups. 

Quaternary ammonium groups, present in Sartobind^®^ Q, remain positively charged across all pH conditions, rendering them strongly basic. In contrast, tertiary amines, as found in Sartobind^®^ D, can exist in a non-ionized form at higher pH levels and become protonated (positively charged) as pH decreases.

For the Sartobind^®^ D at a feed’s pH of 11, an immediate breakthrough occurred, indicating no removal of NOM. For the first sample, a removal was recorded, whereas the second sample had a UV254 value higher than the feed. This can be attributed to the fact that the membranes were pre-flushed with DI water, causing initial dilution of the feed solution. Subsequently, a rapid adsorption of NOM was observed, followed by an immediate desorption. This effect was pronounced due to the alkaline nature of the solution (pH 11) exceeding the IEP of the membrane.

At pH 7 and pH 9, Sartobind^®^ D exhibited nearly identical breakthrough curves, with 50% breakthrough occurring after approximately 220 mL of filtration. The latest breakthrough was recorded when the feed solution had a pH of 4.5, with the breakthrough occurring after approximately 350 mL. Remarkably, after the breakthrough at pH 4.5, there was a substantial removal rate of over 20% even after 500 mL of filtration, compared to less than 10% removal at pH 7 and pH 9. Despite pH 4.5 showcasing the superior performance of the membrane, it is worth noting that the initial removal efficiency was comparatively lower when compared to pH 7 and pH 9. This can be attributed to the general trend for NOM, where, with increasing acidity, there is a reduction in charge density [55]. Consequently, there is a higher proportion of the uncharged NOM fraction, which is not removable by IEX. 

The weakly basic adsorber changes the charge state depending on the pH. Below the IEP, it is protonated and positively charged, making it favorable for the adsorption of negatively charged species in the feed solution. Above the IEP, it becomes deprotonated and uncharged, potentially reducing its adsorption capacity for charged species.

The strong basic adsorber Sartobind^®^ Q, featuring quaternary amines, remains positively charged across all pH conditions (see Figure 4C). However, as the pH increases, the charge density of these positive charges may decrease due to competition with hydroxide ions in the solution. This reduced charge density could lead to a decreased electrostatic attraction between the adsorber and the negatively charged NOM, potentially explaining the decreased performance and earlier breakthrough at higher pH.

### 3.5. pH-Swing Regeneration

**Figure 10:**
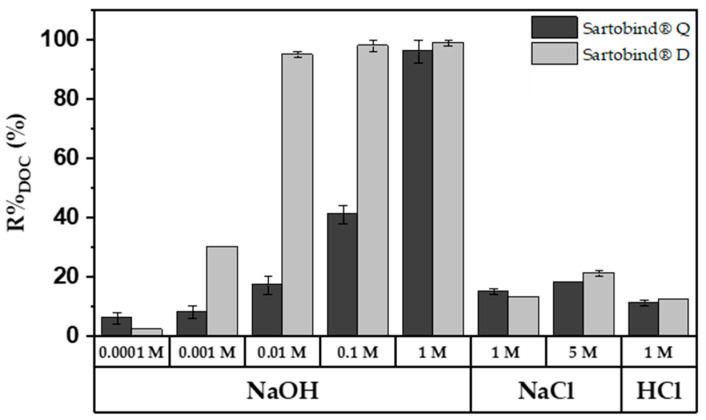
%R_DOC_ for Sartobind^®^ Q and D, utilizing different regeneration solutions: 0.1 mM–1 M NaOH; 1 M NaCl and HCl and 5 M HCl.

The desorption behavior of the membranes was explored using regenerants of varying molarities. Figure 10 displays the regeneration efficiency for previously adsorbed DOC, with 1 M NaOH achieving near-complete regeneration for both membranes. In contrast, 1 M NaCl and HCl achieved only about 15% regeneration. At a lower concentration of 0.01 M NaOH, Sartobind^®^ D showed almost complete regeneration, whereas Sartobind^®^ Q exhibited less than 20% regeneration. At higher concentrations of 0.1 M and 1 M NaOH, regeneration for Sartobind^®^ D did not significantly increase, maintaining high efficiency. However, for Sartobind^®^ Q, regeneration efficiency increased exponentially, reaching over 97% at 1 M NaOH. This indicates a distinct difference in the regenerative response of the two membranes to increasing NaOH concentrations, with Sartobind^®^ Q showing a marked improvement at higher alkali concentrations.

**Figure 11:**
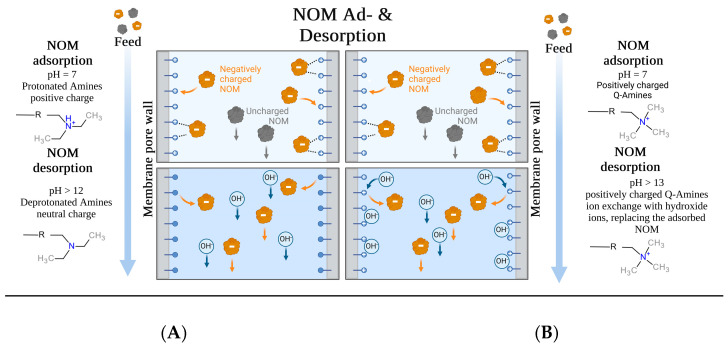
Proposed ad- and desorption mechanisms for (**A**) Sartobind^®^ D and (**B**) Sartobind^®^ Q. (Adapted from “Protein Purification by Hydrophobic Interaction Chromatography—HIC” by BioRender.com (2024). Retrieved from https://app.biorender.com/biorender-templates (accessed on 20 April 2024).

For Sartobind^®^ D, the enhanced desorption with increasing NaOH molarity and consequently higher pH of the regenerant solution underscores that pH-swing-induced deprotonation is the primary mechanism, as supported by the zeta potential data (Figure 4C). This observation is further related to the ongoing deprotonation at pH levels above the IEP of the membrane. Such deprotonation leads to a shift from adsorptive electrostatic attraction to electrostatic repulsion, resulting in the elution and thus regeneration of NOM adsorbed on the membrane. On the other hand, Sartobind^®^ Q possesses an intrinsic charge, yet its regeneration performance improves with increasing molarity due to the presence of hydroxide counter ions. The absence of significant regeneration with HCl and NaCl suggests that the increase in pH modifies the adsorptive characteristics of the membranes, indicating that ion exchange is not the sole mechanism relevant to the regeneration process. Figure 11 presents a proposed ad- and desorption mechanism for both membranes.

### 3.6. Cyclic Adsorption and Regeneration

**Figure 12:**
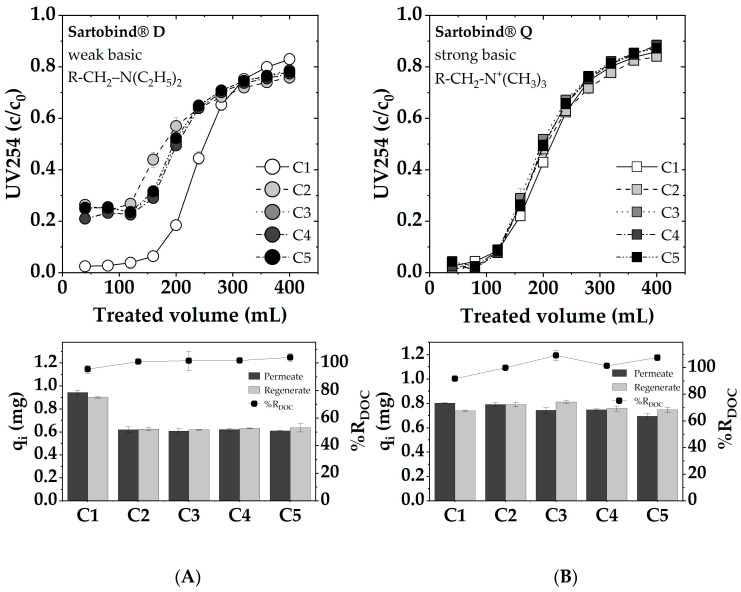
Cyclic adsorption: dynamic breakthrough curves, as represented by the UV 254 absorption, adsorbed and desorbed DOC loadings and %R_DOC_ for (**A**) Sartobind^®^ D and (**B**) Sartobind^®^ Q. Feed: pH 7, DOC: 5 mg/L, UV254: 19.3 m^−1^, 0 mmol/L NaCl, Flux 500 LMH. Regeneration with 1 M NaOH.

In Figure 12, the adsorption breakthrough curves for five consecutive cycles of adsorption and regeneration with 1 M NaOH are presented. The observed performance of Sartobind^®^ Q and Sartobind^®^ D presents a noteworthy contrast. Initially, Sartobind^®^ Q exhibited a slightly earlier breakthrough compared to Sartobind^®^ D, aligning with prior findings. Throughout the five cycles, Sartobind^®^ Q maintained a consistent adsorption performance with only a minor decline in efficiency, indicating stable adsorptive properties and resilience. Although the first cycle showed about 95% regeneration, subsequent cycles achieved nearly complete regeneration, highlighting the effective restoration of adsorptive capacity post-regeneration. In contrast, Sartobind^®^ D displayed a capacity loss and reduced initial removal efficiency after the first cycle, indicating irreversible changes or damage incurred during regeneration with 1 M NaOH. This initial decline stabilized in subsequent cycles, with the performance remaining relatively constant, although at a reduced capacity compared to the initial cycle. Despite the capacity loss in the first cycle, regeneration efficiency for Sartobind^®^ D was remarkably close to 100% across all cycles, suggesting effective removal of adsorbed NOM during the regeneration phase but potentially altered adsorptive sites or affinity post-regeneration. Although the performance metrics provide quantitative insights into the adsorption and regeneration efficiency of the membranes, a photo documentation analysis, as depicted in Table S1, reveals an irreversible coloration on both Sartobind^®^ Q and D after each cycle. This coloration is indicative of some form of fouling, suggesting that, despite the high regeneration efficiencies, some residual compounds or interactions lead to discoloration and potential alteration of the membranes.

The persistent loss in adsorption performance for weakly basic Sartobind^®^ D after initial loading may be explained by structural or chemical changes in the IEX functionalities that are not fully reversible with 1 M NaOH. This phenomenon reflects the fact that restoration of original IEX adsorption capacity is not always possible [56]. To explore potential solutions for mitigating the irreversible capacity loss observed in Sartobind^®^ D after regeneration, additional conditioning experiments were conducted. Two distinct approaches were employed: (1) conditioning with 5 M NaCl and (2) conditioning with 1 M HCl. Conditioning with 5 M NaCl aimed to shift the functional groups into their chloride form, potentially enhancing adsorption performance. However, this treatment did not yield a noticeable improvement, indicating that altering the anion exchange form alone was insufficient to address the irreversible loss in capacity. Conversely, conditioning with 1 M HCl, intended to lower the pH and fully deprotonate the amine groups on the membrane surface, led to almost complete recovery of the initial adsorption performance (see Figure S5). This result suggests that the feed solution’s pH used for filtration, which is pH 7, is not sufficiently acidic to deprotonate all amine sites effectively after regeneration. Therefore, conditioning with acidic HCl post-regeneration is recommended to ensure complete deprotonation and restore the membrane’s original adsorption capacity.

## 4. Conclusions

In summary, the comprehensive IEX membrane adsorber characterization revealed their hydrophilic nature and distinctive surface chemistry. Flux experiments underscored the membrane’s minimal diffusion limitations, endorsing their suitability for dynamic adsorption processes comparatively at high fluxes of up to 500 LMH. Additionally, their effectiveness is evident across a spectrum of feed pH levels and ionic strengths typical of natural waters, although they exhibited distinct behaviors within these parameters. Sartobind^®^ D consistently demonstrated delayed breakthroughs. The adsorption capacities of both membranes were in similar orders with Q_max_ 2888 ± 112 mg C/m^2^ and 2966 ± 153 mg C/m^2^ for Sartobind^®^ D and Q, respectively. Sartobind^®^ D showed notably efficient regeneration with only 0.01 M NaOH. In contrast, Membrane Q necessitated 1 M NaOH for regeneration, highlighting its distinct regenerative requirements. Neither 1 M NaCl, 5 M NaCl nor 1 M HCl proved effective for regeneration, highlighting the relevance of the pH swing deprotonation mechanism for Sartobind^®^ D, in line with zeta potential measurements. The cyclic adsorption study, in which Sartobind^®^ Q showed nearly complete recovery of capacity after regeneration, showcased its resilience. However, Sartobind^®^ D, while fully regenerated, experienced a persistent performance drop after the first cycle, a phenomenon that persisted across subsequent cycles. This behavior underscored the critical role of complete re-protonation in restoring optimal performance after regeneration. Conditioning with 1 M HCl after regeneration emerged as an effective strategy for Sartobind^®^ D’s capacity restoration.

Overall, the present study has shown a proof of concept for the first time that membrane adsorbers are suitable for the removal of NOM from raw waters, and it also has demonstrated promising results within a limited number of cycles. Further studies should investigate into long-term performance and whether membrane adsorbers can also be used to remove other highly relevant organic substances, such as PFAS compounds or micropollutants.

## Figures and Tables

**Table 1:** Membrane properties of Sartobind^®^ D and Q as specified by the manufacturer.

Characteristic	Value
Membrane material	Reinforced stabilized cellulose
Adsorption area/equivalent volume ratio	36.4 cm^2^/mL
Nominal pore size	3–5 µm
Membrane thickness	~250 µm
pH stability	Short term: 2–14 Long term: 2–12
Porosity	~78% [28]

## Data Availability

The original data presented in the study are openly available in Zenodo at DOI: https://doi.org/10.5281/zenodo.10965835.

## Supplementary Materials

The following supporting information can be downloaded at: https://www.mdpi.com/article/10.3390/membranes14060124/s1, Figure S1: Relationship between UV254 and DOC for the SRNOM Isolate 2R101N, dissolved in DI water and filtered through a 0.45 µm filter; Figure S2: Determination of regenerate volume: Regeneration using 1 M NaOH and employing Sartobind® D and Q, with the regenerate carefully pushed through the membrane using a syringe filter. UV254 adsorption of the regenerate is shown as a function of each milliliter collected during the regeneration process; Figure S3: ATR-FTIR spectra of pristine membranes Sartobind^®^ D and Q; Figure S4: EEMs of (A) SRNOM Feed, (B) Permeate Sartobind^®^ D, (C) Permeate Sartobind^®^ Q; Figure S5: Sartobind^®^ D UV254 absorbance curves for cyclic adsorption and regeneration with 1 M NaOH, as well as conditioning with 1 MHCl, DOC sorption and desorption loadings as well as desorption efficiencies for 1 M NaOH regeneration and 1 M HCl conditioning before cycle C6; Table S1: Photo documentation of membranes after regeneration for duplicates D1 and D2, as well as Q1 and Q2, over 5 cycles (C1–C5).
