# Supplementary material for: Characterizing Macroporous Ion Exchange Membrane Adsorbers for Natural Organic Matter (NOM) Removal—Adsorption and Regeneration Behavior
Source: Membranes (Basel). 2024 May 27;14(6):124. doi: 10.3390/membranes14060124 (PMC11205362; doi:10.3390/membranes14060124)
Supplement: Supplementary file 1 [file membranes-14-00124-s001.zip › membranes-2999695-supplementary.pdf]

Supplementary information

Characterizing Macroporous Ion Exchange Membrane Adsorbers for Natural Organic Matter (NOM) Removal—Adsorption and Regeneration Behavior

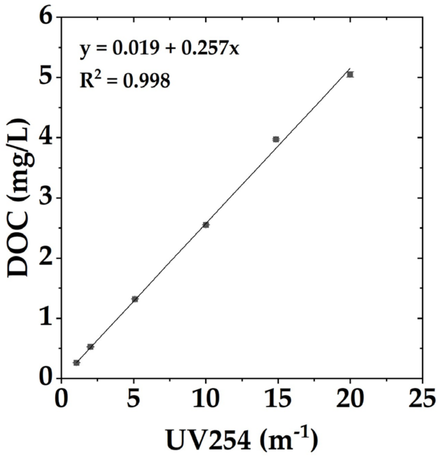

Figure S1. Relationship between UV254 and DOC for the SRNOM Isolate 2R101N, dissolved in DI water and filtered through a 0.45 μm filter.

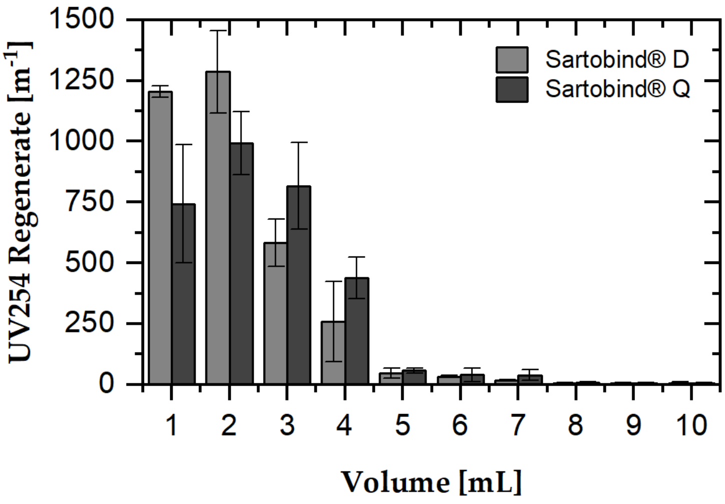

Figure S2. Determination of regenerate volume: Regeneration using 1 M NaOH and employing Sartobind® D and Q, with the regenerate carefully pushed through the membrane using a syringe filter. UV254 adsorption of the regenerate is shown as a function of each milliliter collected during the regeneration process.

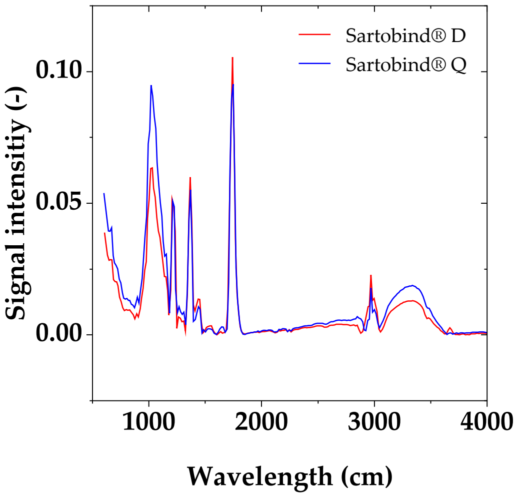

Figure S3. ATR-FTIR spectra of pristine membranes Sartobind® D and Q.

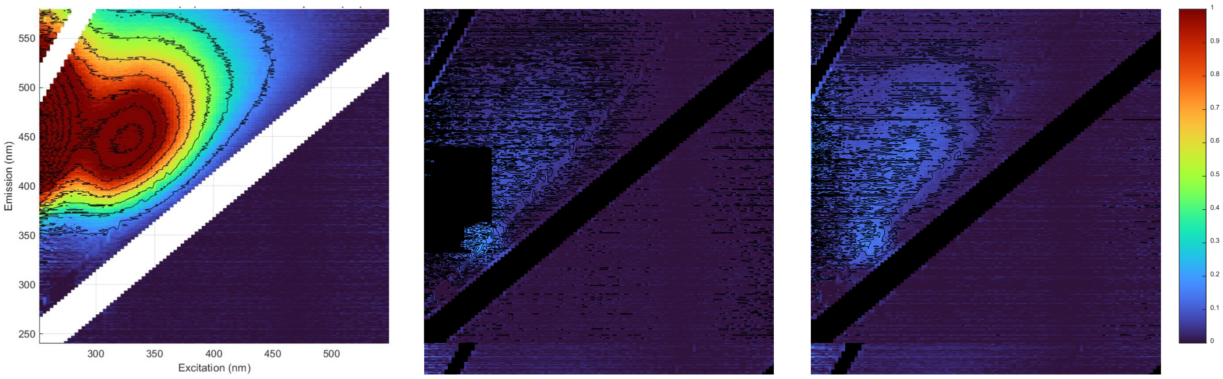

Figure S4. EEMs of (A) SRNOM Feed, (B) Permeate Sartobind® D, (C) Permeate Sartobind® Q.

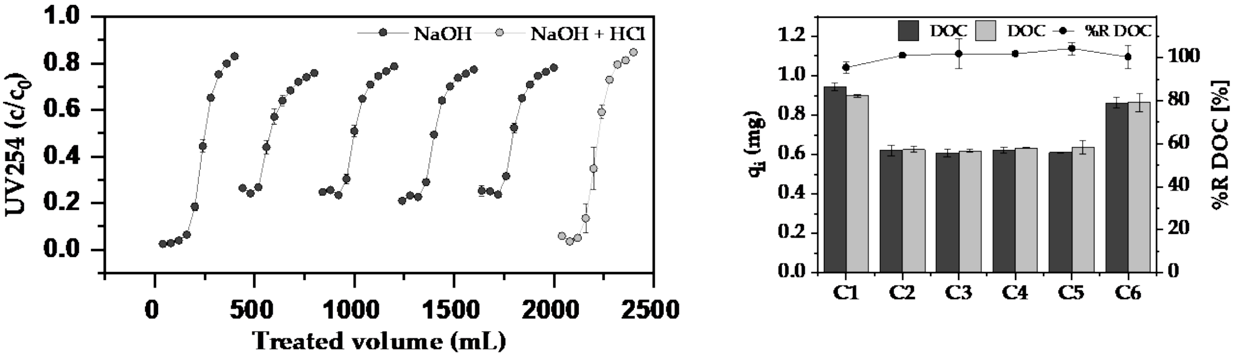

Figure S5. Sartobind® D UV254 absorbance curves for cyclic adsorption and regeneration with 1 M NaOH, as well as conditioning with 1 M HCl, DOC sorption and desorption loadings as well as desorption efficiencies for 1 M NaOH regeneration and 1 M HCl conditioning before cycle C6.

Table S1. Photo documentation of membranes after regeneration for duplicates D1 and D2, as well as Q1 and Q2, over 5 cycles (C1-C5)

**Cycle**	**Experiment**			
	D1	D2	Q1	Q2
C1	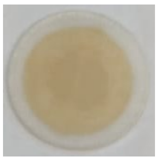	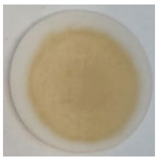	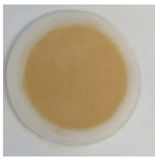	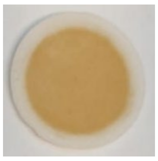
C2	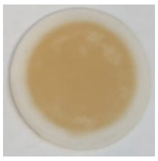	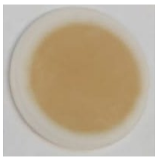	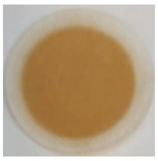	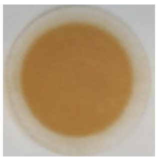
C3	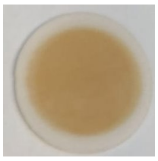	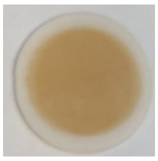	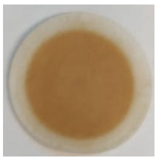	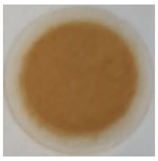
C4	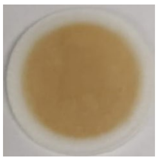	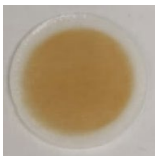	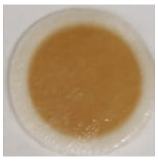	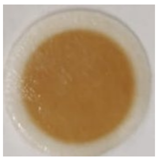
C5	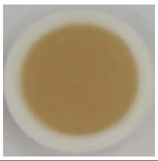	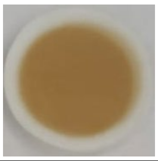	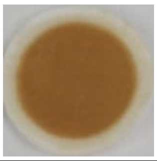	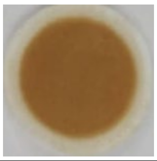
